# Relative Importance of Well-Being Determinants in Atlantic Canadian Families During the Pandemic

**DOI:** 10.1177/00469580231184326

**Published:** 2023-06-27

**Authors:** Taylor G. Hill, Jessie-Lee D. McIsaac, Magdalena Janus, De-Lawrence Lamptey, Melissa D. Rossiter, Joan Turner

**Affiliations:** 1 Dalhousie University, Halifax, NS, Canada; 2 Mount Saint Vincent University, Halifax, NS, Canada; 3 McMaster University, Hamilton, ON, Canada; 4 York University, Toronto, ON, Canada; 5 University of Prince Edward Island, Charlottetown, PE, Canada

**Keywords:** family well-being, pandemic, COVID-19, child health, family leisure, Canada

## Abstract

Framed by the socio-ecological model of well-being, we examined the relative importance of factors contributing to three dimensions of well-being (child, parent, and family) during the COVID-19 pandemic. A sample of 536 participants from the Atlantic provinces of Canada answered a cross-sectional survey in 2021, covering experiences during the pandemic (eg, changes in family life and well-being). Well-being was assessed with 3 single-item measures on positive change in the life of children, parents, and families during the pandemic. This study involved 21 predictor variables (eg, change in time spent on various family activities). Using multiple regression and measures of relative importance based on the Lindeman, Merenda and Gold (lmg) method, we identified the variables most important to predicting well-being. Twenty-one predictors accounted for 21% of the variance in child well-being, 25% in parent well-being, and 36% in family well-being. Well-being at all 3 levels (child, parent, and family) shared the same top predictor (family closeness). The top 6 predictors of well-being at each level were related to leisure (eg, play) and time-use (eg, to prepare meals, engage in self-care, and rest). The effect sizes were smaller for child well-being than at the parent or family level, suggesting there may be important predictors of child well-being not accounted for in these analyses. This study may inform family-level programing and policy that seeks to promote well-being for children and their families.

**While many people experienced challenging and sometimes life-altering situations during**
COVID-19, other people experienced a unique, once-in-a-lifetime opportunity to slow down and consider alternative paths for family life. Positive change that emerge due to the pandemic are largely unexplored. Family cohesion emerged as the most important predictor of well-being for parents, children, and households overall. Leisure (eg, play) and time-use (eg, to prepare meals, engage in self-care, and rest) are also important factors for well-being.
**What do we already know about this topic?**
While many people experienced challenging and sometimes life-altering situations during COVID-19, other people experienced a unique, once-in-a-lifetime opportunity to slow down and consider alternative paths family life.
**How does this research contribute?**
We provide evidence that suggests well-being can be improved by strengthening family cohesion through family leisure time (eg, time playing together, preparing meals, resting).
**What are your research’s implications toward theory, practice, or policy?**
In preparation and planning for potential future pandemics, upstream efforts may be directed toward creating conditions conducive for family closeness (eg, communication and problem-solving skills). Community programing focusing on creating time adequacy for families to play, eat, and rest together may help improve well-being overall.

## Introduction

The global COVID-19 pandemic disrupted daily life routines and established a new normal for individuals, families, and communities. The daily rhythms and previously separated spheres of life (eg, work and play) became blurred. While many people experienced challenging and sometimes life-altering situations that threatened their mental health,^1^ other people experienced a unique, once-in-a-lifetime opportunity to slow down and consider alternative paths for family life. Most evident were changes to the way time was spent, particularly during lockdown and closure periods.^2^ These changes in everyday life provided a welcome re-orientation to some, such as time to spend with others in the household^3^ and find ways to support their own well-being. Identifying the factors that shape well-being for individuals and families during a pandemic is relevant to knowledge generation, policy, and practice.

### Theoretical Framework: Socio-Ecological Model of Well-Being

The influence of all contexts, systems, and environments surrounding an individual and family must be recognized to have a full understanding of, and support, healthy development among individuals and families.^4^ Bronfenbrenner’s ecological systems theory^5,6^ places child development at the center of a complex system of relationships affected by multiple levels of the surrounding environment (ie, immediate settings of family and school all the way to societal influences). A contextual lens helps shift the narrative about mental health and mental illness from an individual issue to the social and environmental responsibility of others^7^ and from a focus on illness to the psychological health and well-being. When attempting to understand the impact of a major societal event, such as the pandemic, this model lends itself to the study of multiple factors influencing children and families.

Families and children are supported by a social ecological system that has been forced to rapidly acclimatize to support families’ needs, often with limited information, during the pandemic. School and child care closures are concerning not only for the interruption to traditional in-person learning, but also for the loss of system-level resources such as nutrition programs, after-school care, school health and counseling services, and school-based vaccination clinics^8,9^ that are positioned to alleviate some consequences of health and social inequities among families considered vulnerable. The first wave of the pandemic showed there were clear risks of returning to high-risk areas (eg, populated spaces, such as the school bus) disproportionally affecting families with lower incomes and fewer resources.^10^ Such conditions may be exacerbated by the placement of child care settings and schools nested within health authorities and government structures that determine many of the policies, services and financial and employment supports available to parents as well as the availability of these supports beyond the pandemic.

Most previous research on well-being has focused on just one or 2 contributing factors, instead of considering the contribution of multiple factors that affect families. Generally, data used to examine well-being has tended to be more economic and health related (and has not included more socio-ecological factors), and regression analyses are typically used to identify important factors in explaining differences in well-being. However, such assessments rarely consider the *relative* importance of the factors. Relative importance refers to the quantification of an individual regressor’s contribution to a multiple regression model^11^ and decomposes overall *R*^2^ into each individual predictor’s contributions. The variance in the outcome accounted for by the predictors is decomposed, with the relative importance of each predictor in the overall *R*^2^ for each possible ordering of predictors is averaged.^12^ Examining relative importance advances the well-being field by enabling researchers to identify what is *most* important. Thus, situated within a socio-ecological approach to well-being, the purpose of this paper is to identify child, parent, and family factors are relatively most important in predicting variations in well-being at the 3 household levels.

### Literature Review

#### Pandemic-related changes and socio-demographic factors

The onset of the pandemic brought restrictions limiting families’ access to spaces outside of their household.^13^ Families with low income who also had low access to outdoor spaces were shown to engage in less self-protection measures (eg, social distancing) than those with higher income and access.^10^ The restrictions also resulted in decreased social spaces through the implementation of gathering limits and restriction of access to shared public spaces. Even though these restrictions may have limited opportunities for in-person social interaction, Tull et al^14^ report that seeking opportunities for social support increased with perceived impacts of the pandemic. That is, reaching out to friends and family was more frequent as the impacts felt greater.

#### Pandemic-related changes in parental experiences

During the pandemic, parents were uniquely burdened with the responsibility of making decisions for their families and children. Most accounts of parental experiences during the pandemic highlight the stressors and challenges experienced during the pandemic. For example, parents reported feeling stressed about a lack of social support and connection,^15-17^ worsened mood and mental health,^18-20^ and challenges managing child behavior.^18,19,21^ Moreover, compared to adults without children, parents experienced more symptoms of anxiety and depression related to the pandemic (eg, experiencing stress over social distancing, the closures of school and childcare, and worrying about the health of others^15^).

#### Positive change in family life due to pandemic

At present, much of the literature reports little on the potential for positive experiences during the pandemic. For example, in one study, less than one-third of parents reported no positive experiences while the two-thirds of parents spoke of benefits such as moving at a slower pace, feeling grateful, and spending more time together as a family.^15^ Some families experience unexpected improvements and resources such as strengthened parent, child, and sibling relationships and psychological adaptiveness.^22^ The most commonly reported positive changes to family life was an increase in family time,^10,21,23-25^ including increased time for parents and children to play together,^26,27^ and for specific activities such as playing board games, arts and crafts, and bike riding.^24^ For some families, reduced employment hours during the pandemic had a positive impact on their family environments despite salary reduction.^17^ Families also reported more time to prepare and eat meals together.^22^ Some parents reported an increase in family time and affection shown to one another.^20^ Increased time spent with family may also serve as a resource for parental well-being; Janssen et al^16^ found that parents perceived spending time with family such as cooking and watching television together to be helpful for family connection during lockdown. The fact that the actual time spent in these activities was not measured before and after the pandemic makes the quantitative interpretation of these results challenging; a challenge that is mitigated by asking about perceptions of change. In our study of positive contributors, we also used the perceived change due to the pandemic, as the outcome measure.

### Rationale and Objective

Most studies highlight challenges that families faced in responding to pandemic restrictions and leave opportunities for a positive change that emerge due to the pandemic largely unexplored, even those these may be factors that buffer the impact of the pandemic on well-being. The purpose of this paper is to identify pandemic-related factors that contribute to the well-being of children (ages up to 8 years to focus on period of early childhood), parents, and family.^28^

### Research Questions

1: Which pandemic-related factors are most important for change in child well-being?
2: Which pandemic-related factors are most important for change in parent well-being?
3: Which pandemic-related factors are most important for change in family well-being?

## Method

All survey materials and data analysis syntax are posted online on our Open Science Framework page: https://osf.io/q6h8y/?view_only=5d3c0cffa9064ebbb4f3f7ecb1ff437d.

### Participants

Any family with a child 0 to 8 years residing in the Atlantic Provinces of Canada, that is, Nova Scotia, Prince Edward Island, New Brunswick, and Newfoundland, during the pandemic were eligible to take part in this survey. The sample (N = 536) was 79% women and with a median annual household income of greater than $100 000 (see Table 1). Potential participants were recruited via a poster shared with relevant family-focused organizations (eg, child care, family resource) in the Atlantic provinces and extensive social media campaign. The survey was administered online through Simple Survey due to pandemic measures. The survey was available online from March 9th 2021 to April 5th 2021.

**Table 1.** Descriptive Statistics of Survey Variables.

Variable	*M* (SD)	*%*
Province		
New Brunswick		20.99
Nova Scotia		46.09
Prince Edward Island		11.66
Newfoundland and Labrador		21.26
Lives in a rural community		15.50
Parenting arrangement		
Single parent household		6.45
Dual parent household		86.97
Dual parents, two households		4.66
Other parenting arrangement		1.18
Parent is an essential worker		86.97
Parent lives with a disability		4.13%
Annual household income level in Canadian dollars		
Less than 20 000		2.60
21 000 to 40 000		7.36
41 000 to 60 000		9.04
61 000 to 80 000		10.11
81 000 to 100 000		16.39
More than 100 000		44.10
Parent’s highest education level		
High school		5.84
Community or technical college		28.64
Undergraduate degree		29.10
Graduate degree		35.99
Number of children		
1		35.66
2		46.91
3		11.93
4		3.56
5 or more		1.5
Age of children		
Under 12 months		11.11
12 to 18 months		9.05
19 to 35 months		25.79
3 to 5 years		60.91
6 to 8 years		37.86
9 to 12 years		17.42
Over 13 years		7.68
Job Status of Parent		
Full time		74.07
Part time		9.87
On leave		7.81
Work from home		8.5
Student		4.53
Unemployed		1.78
Unable to work		0.1
Gender of Parent		
Man		9.47
Woman		79.29
Transgender		10.42
Ethnicity of Parent		
Indigenous		7
Acadian		16.87
European		57.34
African		1.37
Middle Eastern		1.10
Latin America		0.1
Asian		2.51
Other ethnicity (not listed)		5.35
Parent perceptions of own change from COVID-19 (Range 1 to 5)		
Feeling worried	3.81 (1.11)	
Concerned with ability to manage child’s emotional well-being	2.97 (0.92)	
Concerned with ability to manage child’s behavior	3.06 (1.12)	
Feeling disconnected from my friends/family	3.82 (1.19)	
More comfortable supporting child’s play	3.07 (0.97)	
More time to prepare healthy meals	2.67 (1.10)	
More time to take care of self	2.10 (1.08)	
Feeling more rested	2.02 (0.94)	
Parent perceptions of family change from COVID-19 (Range 1 to 5)		
Relationship strength	2.59 (1.11)	
Tension among family members	3.12 (1.15)	
More time to spend outdoors	2.96 (0.92)	
Family spends time using screens alone	3.56 (1.00)	
Family eats meals together	3.29 (0.94)	
Family cooks together	3.37 (0.95)	
Family reads together	3.27 (0.91)	
Family plays together	3.55 (0.90)	
Parent perceptions of child change from COVID-19 (Range 1 to 5)		
Child has more consistent mealtime and snack routines	2.92 (0.93)	
Child spends time using screens alone	3.66 (1.17)	
Child takes part in more energetic play	2.88 (0.91)	
Child plays outside more	3.28 (0.98)	
Child plays alone more	3.56 (1.10)	
Child has easier sleep routines	2.77 (0.93)	
Child spends more time with family	4.04 (1.00)	
Parent perceptions of child’s mood from COVID-19 (Range 1 to 3)		
Child feels happy	1.94 (0.52)	
Child feels lonely	1.51 (0.52)	
Child feels worried	1.54 (0.53)	
Overall change as a result of COVID-19 (Range 1-4)		
Child—positive change	2.06 (0.74)	
Child—negative change	2.70 (0.72)	
Parent—negative change	2.72 (0.75)	
Parent—positive change	2.08 (0.79)	
Family—positive change	2.28 (0.75)	
Family—negative change	2.60 (0.73)	

#### Survey instrument

Building on the authors’ research early in the pandemic, the survey included similar closed and open-ended questions to explore changing family experiences and emerging issues related to well-being identified. The authors with expertise in early childhood development research developed the survey in collaboration with government and local health authorities to improve validity of the survey in the context of the pandemic. The preliminary survey was piloted among families with children aged 0 to 8 years in the Atlantic provinces and the results informed the final survey used in this study to improve validity and reliability. The survey was comprised of 3 major sections. The first included questions about the child(ren) in the family, for example, health condition, parenting arrangements, isolation due to the pandemic, and caregivers at home or in child care. The second major section gathered information on the adults in the household, including working conditions, partner demographics, workplace changes, social distance adherence, and changes in family life. Finally, the third section included items related to living standards, such as food security, accessing to services and supports, resilience, attitudes toward the pandemic, and demographic characteristics.

### Measures

#### Well-being

Positive changes in well-being due to the pandemic were measured with three single 4-point items. The child well-being measure asked “How would you rate the overall level of positive change in your child(ren) as a result of the pandemic?.” The parent well-being measure asked “How would you rate the overall level of positive change in your own life as a result of the pandemic?.” Finally, the parent well-being measure asked “How would you rate the overall level of positive change in your family life as a result of the pandemic?” Response options to all 3 items were: 1 = none, 2 = minimal, 3 = moderate, 4 = extreme.

Even though the reliability and validity of single-item well-being measures have been challenged, research suggests they are psychometrically sound,^29,30^ and they are certainly effective for inclusion in multi-purpose surveys. Cheung and Lucas & Lucas^31^ showed that single-item well-being measures perform similarly to multiple-item well-being scales and that single-item measures did not produce systematically different correlations compared to multiple-item well-being measures on theoretically relevant variables. The reliability of single-item measures has been deemed moderate to acceptable.^32-35^

#### Demographic variables

Thirteen demographic variables were included in the analysis. *Age of child(ren)* was measured as a categorical variable, where parents could report more than one age group if they had more than one child in a different age group (ie, less than twelve months = 1, twelve to eighteen months = 2, nineteen to thirty-five months = 3, three to five years = 4, six to eight years = 5, nine to twelve years = 6, over 13 years old = 7). *Annual household income* was measured using 6 groupings ranging from less than $20 000 to $100 000 and higher. *Highest education level completed* was to be reported for anyone in the family and measured using 5 groupings starting with junior/middle school and ending with graduate degree. *Province* was measured as a categorical variable (ie, New Brunswick = 1, Nova Scotia = 2, Prince Edward Island = 3, Newfoundland and Labrador = 4). Parenting arrangement was measured as a categorical variable (ie, single parent = 1, two parents living together in the same home = 2, two or more parents living separately in 2 or more homes = 3, non-parent primary caregiver = 4). *Gender identity* was measured as a categorical variable (ie, man = 1, woman = 2, cultural (eg, 2-spirit) = 3, non-binary = 4, transgender = 5, other = 6). *Ethnicity* was measured as a categorical variable (ie, Indigenous = 1, Acadian = 2, European = 3, African = 4, Middle-East = 5, Asian = 6, Latin America = 7, other/not listed = 8). *Number of children* was measured as a continuous variable. *Job status* was measured as a categorical variable (part-time = 1, full-time = 2, parental leave = 3, work from home = 4, student = 5, unemployed = 6, unable to work = 7). Other demographic variables included were dichotomous and measured as binary variables: *rurality* (ie, urban = 0 or rural = 1), *essential worker* (ie, whether the parent was considered an essential worker in their province; essential = 1 or not = 0), *disability status*, (ie, identifies as someone with a disability = 1 or not = 0).

*Food security* was measured as a categorical variable using the United States Household Food Security Survey Module: Six Item Short Form^36^ which was developed by researchers at the National Center for Health Statistics. Participants read “These next questions are about the food eaten in your household in the last 12 months (since May of last year), and whether you were able to afford the food you need” and answered 6 questions regarding the frequency with which they experienced a threat to their food security (eg, “In the last 12 months, were you ever hungry but didn’t eat because there wasn’t enough money for food?”). The scale is scored to identify 3 levels of food security (scores x-y high or marginal food security, x-y low food security, and x-y very low food security).

#### Child-level measures

Participants (parents) answered 7 questions about changes in their child(ren)’s life due to the pandemic on a 5-point Likert-type scale (strongly disagree = 1, strongly agree = 5). These questions included: a) more consistent mealtime and snack routines; b) spends time using screens alone; c) takes part in more energetic play; d) plays outside more; e) plays alone more; f) has easier sleep routines; and g) spends more time with family. Three additional questions asked parents to identify the presence and/or type of change in their child in terms of mood on a three-point scale (decreased = 1, no change = 2, increased = 3): a) happiness; b) loneliness; and c) worry. Two final questions asked parents to identify level of positive and negative change in their child overall on a four-point scale (no change = 0, minimal = 1, moderate = 2, extreme = 3).

#### Parent-level measures

Participants (parents) answered 8 questions about changes in their own life due to the pandemic on a 5-point Likert-type scale (strongly disagree = 1, strongly agree = 5). There questions included: a) feeling worried; b) concerned with ability to manage child’s emotional well-being; c) concerned with ability to manage child’s behavior; d) feeling disconnected from friends/family; e) more comfortable supporting child’s play; f) more time to prepare healthy meals; g) more time to take care of self; and h) feeling more rested. Parents were also asked to identify level of overall positive and negative change in their own life on a four-point scale (no change = 0, minimal = 1, moderate = 2, extreme = 3).

#### Family-level measures

Participants (parents) answered 8 questions about changes in their family due to the pandemic on a 5-point Likert-type scale (strongly disagree = 1, strongly agree = 5). These questions included: (a) relationship strength; (b) tension among family members; (c) more time to spend outdoors; (d) more time using screens alone; (e) eating meals together; (f) cooking together; (g) reading together; and (h) playing together. Parents were also asked to identify level of overall positive and negative change in their family on a four-point scale (no change = 0, minimal = 1, moderate = 2, extreme = 3).

### Data Analysis Plan

Data were analyzed using R (version 4.0.5). Multiple linear regression was used to test predictors of positive change in ordinal scales in children, in parents, and in the family, in separate models. Given the large number of potential predictors in the dataset, identifying the relative importance of each predictor is more informative than relying on traditional null hypothesis significance testing metrics. Relative importance refers to the quantification of an individual regressor’s contribution to a multiple regression model^11^ and decomposes overall *R*^2^ into each individual predictor’s contributions, with the relative importance of each predictor in the overall *R*^2^ for each possible ordering of predictors averaged.^12^ For effect sizes, we relied on measures of relative importance using the Lindeman, Merenda and Gold (lmg) method in Groemping, (2007) relaimpo() package in R. Relative importance is a decomposition of the total *R*^2^ for each variable such that coefficients sum to *R*^2^; in other words, relative importance is the proportion of the total *R*^2^ contributed by each predictor.

## Results

### Preliminary Data Analysis

**Figure 1.**
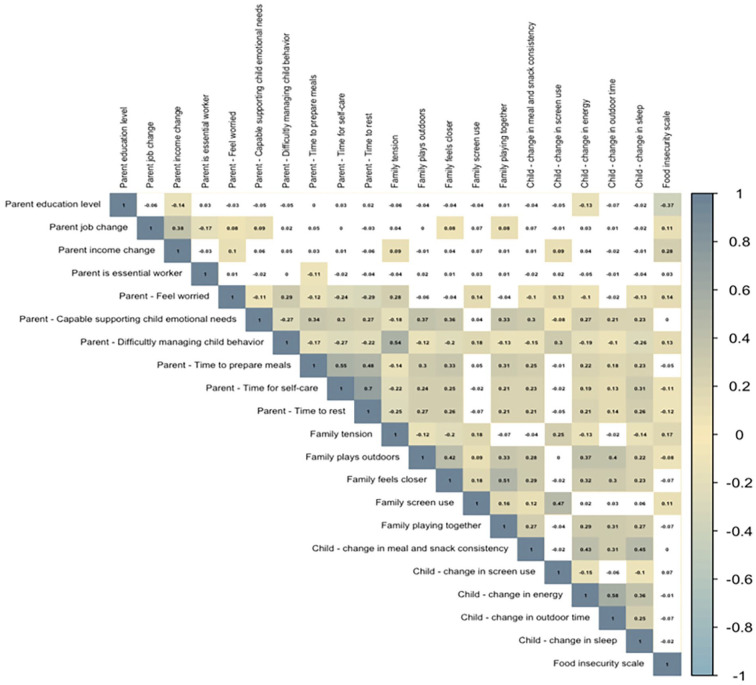
Correlation matrix of continuous survey variables. Insignificant correlates are in white cells.

Nearly half of the respondents were from Nova Scotia (45%), nearly one quarter from New Brunswick (23%), with the remaining from Newfoundland (21%), and Prince Edward Island (11%). Bivariate correlations between key study variables are presented in Figure 1. In general, the predictor variables were moderately correlated with each other as one would expect and none of the correlations were strong enough to cause concern over multicollinearity and as such could be assessed for their relatively unique contributions to well-being.

### Primary Data Analysis

#### Model 1. Multiple regression predicting child(ren) well-being

Our first regression model was built to predict child well-being based on 21 independent variables (see Table 2). We used relative importance (ri) to identify which variables predicted the most variance in the outcome.

**Table 2.** Multiple Regression Model Predicting Child Well-Being.

Coefficient	Estimates	Std. Beta	Conf. Int (95%)	standardized CI	*P*-value
Parent education level	0.01	0.01	–0.05 to 0.07	–0.07 to 0.09	.776
Parent job change	0.20	0.10	0.04 to 0.36	0.02 to 0.18	**.012**
Parent income change	–0.02	–0.02	–0.15 to 0.10	–0.10 to 0.07	.697
Parent is essential worker	0.10	0.07	–0.01 to 0.21	–0.00 to 0.14	.067
Parent—Feel worried	–0.03	–0.04	–0.08 to 0.02	–0.12 to 0.04	.290
Parent—Capable supporting child emotional needs	0.07	0.09	–0.00 to 0.14	–0.00 to 0.17	.051
Parent—Difficultly managing child behavior	–0.04	–0.07	–0.11 to 0.02	–0.16 to 0.03	.184
Parent—Time to prepare meals	0.03	0.04	–0.04 to 0.09	–0.05 to 0.13	.405
Parent—Time for self-care	0.05	0.07	–0.03 to 0.12	–0.05 to 0.18	.264
Parent—Time to rest	0.02	0.03	–0.06 to 0.11	–0.08 to 0.14	.592
Family tension	–0.03	–0.05	–0.09 to 0.03	–0.14 to 0.04	.314
Family plays outdoors	–0.04	–0.05	–0.11 to 0.03	–0.14 to 0.04	.274
Family feels closer	0.13	0.16	0.05 to 0.21	0.06 to 0.25	**.001**
Family screen use	–0.05	–0.07	–0.11 to 0.01	–0.15 to 0.02	.125
Family playing together	0.07	0.08	–0.01 to 0.14	–0.01 to 0.17	.076
Child—change in meal and snack consistency	–0.03	–0.04	–0.10 to 0.04	–0.13 to 0.05	.375
Child—change in screen use	0.05	0.08	–0.01 to 0.10	–0.01 to 0.16	.081
Child—change in energy	0.13	0.15	0.04 to 0.21	0.05 to 0.25	**.003**
Child—change in outdoor time	0.03	0.04	–0.04 to 0.10	–0.05 to 0.14	.383
Child—change in sleep	0.10	0.12	0.03 to 0.17	0.04 to 0.21	**.006**
Food insecurity	–0.02	–0.08	–0.05 to 0.00	–0.17 to 0.00	.053
*N*	556				
*R* ^2^	0.28				

*Note*.*P*-values are bolded to indicate significance at the *P* < .05 level.

Collectively, the 21 variables contributed nearly one-third of the variance in well-being (*R*^2^ = 0.28), mainly due to the relative importance of 6 variables (*R*^2^ = 0.16): family closeness (ri = 0.04), child energy level (ri = 0.03) and sleep quality (ri = 0.03), family playing together (ri = 0.03), parent’s perceived capacity to support children’s emotional needs (ri = 0.02), and having time for self-care (ri = 0.02).

#### Model 2. Multiple regression predicting parent well-being

In order to examine change in parent well-being, our second regression model was built from the same 21 independent variables as above (see Table 3).

**Table 3.** Multiple Regression Model Predicting Parent Well-Being.

Coefficient	Estimates	Std. Beta	Conf. Int (95%)	standardized CI	*P*-value
Parent education level	0.02	0.02	–0.05 to 0.08	–0.05 to 0.09	.601
Parent job change	0.12	0.06	–0.04 to 0.28	–0.02 to 0.13	.127
Parent income change	–0.03	–0.02	–0.16 to 0.10	–0.09 to 0.06	.656
Parent is essential worker	–0.03	–0.02	–0.15 to 0.08	–0.09 to 0.05	.552
Parent—Feel worried	–0.07	–0.09	–0.12 – –0.01	–0.17 – –0.02	**.015**
Parent—Capable supporting child emotional needs	0.02	0.03	–0.05 to 0.09	–0.06 to 0.11	.538
Parent—Difficultly managing child behavior	–0.02	–0.03	–0.09 to 0.04	–0.12 to 0.06	.470
Parent—Time to prepare meals	0.13	0.18	0.07 to 0.19	0.09 to 0.27	<**.001**
Parent—Time for self-care	0.08	0.11	0.00 to 0.16	0.00 to 0.22	**.044**
Parent—Time to rest	0.07	0.09	–0.01 to 0.16	–0.01 to 0.19	.092
Family tension	–0.06	–0.08	–0.12 to 0.00	–0.17 to 0.00	.054
Family plays outdoors	–0.07	–0.08	–0.14 to 0.01	–0.16 to 0.01	.070
Family feels closer	0.18	0.21	0.11 to 0.26	0.12 to 0.30	<**.001**
Family screen use	–0.05	–0.06	–0.11 to 0.02	–0.14 to 0.02	.162
Family playing together	0.07	0.07	–0.01 to 0.14	–0.01 to 0.16	.082
Child—change in meal and snack consistency	–0.02	–0.02	–0.09 to 0.05	–0.11 to 0.06	.600
Child—change in screen use	0.00	0.01	–0.05 to 0.06	–0.08 to 0.09	.877
Child—change in energy	0.03	0.03	–0.06 to 0.11	–0.06 to 0.12	.542
Child—change in outdoor time	0.07	0.09	–0.00 to 0.14	–0.00 to 0.17	.060
Child—change in sleep	0.04	0.05	–0.03 to 0.11	–0.04 to 0.13	.281
Food insecurity	–0.00	–0.02	–0.03 to 0.02	–0.09 to 0.06	.699
*N*	556				
*R* ^2^	0.37				

*Note*. *P*-values are bolded to indicate significance at the *P* < .05 level.

Collectively, the 21 variables predicted more than one-third of the variance in well-being (*R*^2^ = 0.37), mainly due to the relative importance of 6 variables (*R*^2^ = 0.25): family closeness (ri = 0.06), parent having time to prepare meals (ri = 0.06), engage in self-care (ri = 0.05), to rest (ri = 0.04), to play (ri = 0.03), and lowered family tension (0.02).

#### Model 3. Multiple regression predicting family well-being

Our third regression model was built to predict family well-being from the same 21 independent variables as above (see Table 4).

**Table 4.** Multiple Regression Model Predicting Family Well-Being.

Coefficient	Estimates	Std. Beta	Conf. Int (95%)	standardized CI	*P*-value
Parent education level	–0.00	–0.00	–0.06 to 0.06	–0.08 to 0.07	.941
Parent job change	0.11	0.05	–0.04 to 0.26	–0.02 to 0.13	.157
Parent income change	–0.07	–0.04	–0.19 to 0.05	–0.12 to 0.03	.266
Parent is essential worker	–0.03	–0.02	–0.14 to 0.07	–0.09 to 0.05	.537
Parent—Feel worried	–0.05	–0.07	–0.10 to 0.01	–0.14 to 0.01	.078
Parent—Capable supporting child emotional needs	0.08	0.10	0.01 to 0.15	0.02 to 0.18	**.019**
Parent—Difficultly managing child behavior	–0.01	–0.01	–0.07 to 0.06	–0.10 to 0.08	.848
Parent—Time to prepare meals	0.06	0.09	0.00 to 0.13	0.00 to 0.18	**.040**
Parent—Time for self-care	0.01	0.01	–0.07 to 0.08	–0.10 to 0.12	.886
Parent—Time to rest	0.07	0.09	–0.01 to 0.16	–0.01 to 0.19	.091
Family tension	–0.06	–0.09	–0.12 – –0.00	–0.18 – –0.01	**.037**
Family plays outdoors	–0.05	–0.07	–0.12 to 0.01	–0.15 to 0.02	.120
Family feels closer	0.20	0.24	0.13 to 0.28	0.15 to 0.33	<**.001**
Family screen use	–0.07	–0.09	–0.13 to –0.00	–0.17 to –0.01	**.036**
Family playing together	0.19	0.23	0.12 to 0.26	0.15 to 0.32	<**.001**
Child—change in meal and snack consistency	–0.02	–0.03	–0.09 to 0.04	–0.11 to 0.05	.496
Child—change in screen use	0.02	0.03	–0.03 to 0.07	–0.05 to 0.12	.426
Child—change in energy	–0.05	–0.05	–0.13 to 0.03	–0.15 to 0.04	.266
Child—change in outdoor time	0.05	0.07	–0.02 to 0.12	–0.02 to 0.16	.135
Child—change in sleep	0.06	0.08	–0.00 to 0.13	–0.01 to 0.16	.067
Food insecurity scale	–0.00	–0.01	–0.03 to 0.02	–0.09 to 0.07	.871
*N*	557				
*R* ^2^	0.36				

*Note*. *P*-values are bolded to indicate significance at the *P* < .05 level.

Collectively, the 21 variables predicted over one-third of the variance in well-being (*R*^2^ = 0.36), mainly due to the relative importance of 6 variables (*R*^2^ = 0.26): family closeness (ri = 0.08), family time to play (ri = 0.08) and prepare meals (ri = 0.03), parent’s perceived capacity to support children’s emotional needs (ri = 0.03), parent having time to rest (ri = 0.03) and engage in self-care (0.02).

A comparison of relative importance between the models predicting variance in change in well-being in children, parents and family is presented in Table 5, showing the 3 measures of well-being share the same top predictor (family closeness). Of note, the top predictors (those individually accounting for at least 2% of the variance in the outcome) accounted for less variance in child well-being than in parent or family well-being. In addition, a small squared semi-partial correlation (despite a relatively large relative importance) for any variable suggests that the predictor variable is not directly related to well-being, but rather, overlaps strongly with many of the other predictors (see Table 5)

**Table 5.** Comparison of Relative Importance of Independent Variables Predicting Well-Being of Atlantic Households, Top Predictors (ri ≥ 0.02) Are Bolded.

Child well-being		Parent well-being		Family well-being	
**Family feels closer**	**0.041**	**Family feels closer**	**0.058**	**Family feels closer**	**0.079**
**Child**—**change in energy**	**0.033**	**Parent**—**Time to prepare meals**	**0.057**	**Family plays together**	**0.077**
**Child**—**change in sleep**	**0.025**	**Parent**—**Time for self-care**	**0.046**	**Parent**—**Time to prepare meals**	**0.029**
**Family plays together**	**0.025**	**Parent**—**Time to rest**	**0.043**	**Parent**—**Can support child emotional needs**	**0.028**
**Parent**—**Can support child emotional needs**	**0.020**	**Family plays together**	**0.026**	**Parent**—**Time to rest**	**0.026**
**Parent**—**Time for self-care**	**0.020**	Family tension	**0.020**	**Parent**—**Time for self-care**	**0.020**
Parent—Difficultly managing child behavior	0.017	Parent—Feel worried	0.017	Family tension	0.019
Parent—Time to rest	0.016	Parent—Difficultly managing child behavior	0.015	Child—change in sleep	0.015
Child—change in outdoor time	0.015	Parent—Can support child emotional needs	0.015	Child—change in outdoor time	0.011
Parent—Time to prepare meals	0.014	Child—change in outdoor time	0.014	Parent—Difficultly managing child behavior	0.011
Family tension	0.012	Child—change in sleep	0.013	Parent—Feel worried	0.009
Food insecurity scale	0.009	Child—change in energy	0.013	Family plays outdoors	0.008
Child—change in meal/snack consistency	0.008	Family plays outdoors	0.007	Child—change in energy	0.007
Parent job change	0.008	Child—change in meal/snack consistency	0.006	Child—change in meal/snack consistency	0.006
Family plays outdoors	0.007	Food insecurity scale	0.004	Parent job change	0.004
Parent—Feel worried	0.006	Parent job change	0.004	Family screen use	0.003
Parent is essential worker	0.003	Family screen use	0.003	Food insecurity scale	0.002
Child—change in screen use	0.002	Child—change in screen use	0.002	Child—change in screen use	0.001
Family screen use	0.002	Parent is essential worker	0.002	Parent is essential worker	0.001
Parent education level	0.001	Parent education level	0.001	Parent income change	0.001
Parent income change	0.000	Parent income change	0.000	Parent education level	0.000
*R*^2^ of **top predictors**	**0.16**	*R*^2^ of **top predictors**	**0.25**	*R*^2^ of **top predictors**	**0.26**
*R*^2^ (total)	0.28	*R*^2^ (total)	0.37	*R*^2^ (total)	0.36

## Discussion

The purpose of this paper was to identify which pandemic related factors are the strongest contributors to the change in well-being in children, parents, and families during a pandemic. Given the number of potential predictors in the dataset, identifying the relative importance of each predictor is more informative than relying on traditional null hypothesis significance testing metrics. We analyzed the relative importance of dozens of predictor variables to predict as much variance in well-being as possible. Well-being at all 3 levels (child, parent, and family) shared the same top predictor (family closeness), although the effect sizes were smaller for child well-being than at the parent or family level, suggesting there may be important predictors of child well-being during a pandemic not accounted for in these analyses. In the following section, we focus on unpacking the top predictors of variation in well-being shared between all levels, in order of their relative importance (ie, family closeness and then increased time playing together, preparing meals, resting, and engaging in self-care).

### Family Closeness and Relationships

At each level (ie, child, parent, family) family closeness and relationships were the strongest predictor of well-being in this study. For children, parents, and the family overall, feelings of belonging to a close-knit family unit was the most important factor for a sense of well-being. Aligned with previous research, families may experience unexpected improvements and resources during the pandemic, such as strengthened parent, child, and sibling relationships and psychological adaptiveness.^22^ Some parents have identified pandemic-related benefits of moving at a slower pace, feeling grateful, and spending more time together as a family,^15^ all of which may contribute to well-being. In Canada, many parents reported increased positive interactions at home (particularly those stressed about financial concerns or with pre-existing mental health conditions^37^) which can include feelings of family closeness, an important determinant of well-being.

Previous research shows parents reported having more quality time together, feeling closeness in the family, showing love and affection, and observing resilience in their children.^37^ For example, Gadermann et al^37^ found that parents reported increases in both negative and positive interactions with children due to the COVID-19 pandemic, possibly due to increased opportunities for family interactions overall during lockdown and closure periods. Although families may experience increased stress and worry, some parents reported an increase in conflict that coincided with an increase in family time and affection shown to one another.^20^ These increased opportunities for family interactions may act as a pre-cursor to feelings of family closeness. For example, some scholars suggest that increased time and flexibility at home has created conditions for families to engage in more conversations and activities together.^38^ When families spend time together, there is an increased opportunity to bond, problem solve, and ultimately becoming closer,^39^ which is important to well-being. Although parenting pressures during the pandemic have increased,^40^ so have opportunities to strengthen family closeness and well-being.

### Adequate Time to Rest, Prepare Meals, Self-Care, and Play Together

Our findings show that well-being is improved when families play together and when parents have time to prepare meals, eat, rest, and engage in self-care. Other research has shown the most commonly reported positive changes to family life as a result of the pandemic was an increase in time spent together as a family,^10,21,23-25^ regardless of time-use activities. The time affluence reported in the literature included increased leisure time for parents and children to play together^26,27^ on activities such as playing board games, arts and crafts, and bike riding.^24^ According to our study and previous studies, this time spent together is important for well-being. A main cause for panic during the pandemic was the changes to the routine comfort of daily life^41^ which people are familiar with. Zhang^38^ recommended parents try to maintain children’s daily life rhythms (ie, work and rest balance, regular activities) with a focus on adequate daily activities such as reading, indoor sports, games, and handicrafts rather than paying too much attention to information about the pandemic. For example, Janssen et al,^16^ found that parents perceived spending time with family such as cooking and watching television together to be helpful during lockdown. Hood et al,^24^ found families reported having more time for playing board games, arts and crafts, and bike riding. This “gamification” of leisure time provides a variety of activities and creation of spaces of fun and flow,^42^ which could serve as a home-based well-being promotion tool for families.

Previous research suggested a link between satisfaction with family leisure and overall satisfaction with family life.^39^ The increased opportunity for families to bond may include strengthening communication skills within family dynamics,^43^ developing problem solving skills,^44^ and generally increasing satisfaction with family life.^45^ Different types of leisure require different levels of engagement.^46^ Families spending time together with little interaction (eg, watching television) is known as parallel family leisure. On the other hand, a joint activity would be a family dinner which provides the opportunity for family members to talk and share their thoughts with each other. Both types of leisure activities (parallel and joint) were top predictors of well-being in the current study. Families’ participation in leisure time has been linked to family cohesion, family adaptability, and overall family functioning^47-49^ three dimensions of family well-being that are critical to being resilient during a pandemic.

### Limitations and Future Directions

The contextual factors of the Atlantic provinces may limit the degree to which findings can be applied to other locations (eg, Nova Scotia’s rurality, Newfoundland’s higher rates of poverty, and a region-specific early childhood system). The cross-sectional time-limited surveys are limited in the type of research questions that can be addressed (as no interactive follow-up is possible based on the respondent answer], due to the focus on one point in time. Although one-time surveys are efficient in gathering and interpretation of quantitative data, especially in the face of special events such as the COVID-19 pandemic, they are limited in their ability to probe responses. Survey respondents were disproportionately female, meaning that all information is limited to the perspective of the mother in the household. Incorporating the voice of the child(ren) or a second parent would fit with the systems-level perspective we take, such as understanding the results in the context of family dynamics. The cross-sectional design of the study made it impossible to examine longitudinal trends in family well-being and pandemic-related changes or examine long-term outcomes of the pandemic and of family closeness. In the future, improving study design, such as using longitudinal methods or mixed-methods, would be beneficial to gaining a richer understanding of families’ experiences and well-being during the pandemic. Survey questions would also benefit from further validation studies and including a neutral mid-point option on the scale, to prevent forcing respondents to answer while mitigating problems arising from missing data. Finally, this study provided insight on the well-being of children during the pandemic from their caregivers’ perspective, although it was limited to children aged 0 to 8 years. Future research is needed to explore the experiences of older children and their families.

## Conclusion

Due to the COVID-19 pandemic, the social ecological system supporting children and families has been forced to quickly change to support families’ needs. A silver lining emerging from the pandemic is the positive changes to family life, such as an increase in time spent together. Our study found that child, parent, and family well-being shared the same top contributing factor, namely family closeness. The remaining top predictors of well-being were related to family leisure time, such as increased time playing together, preparing meals, resting, and engaging in self-care, which may inform programing and policy that seeks to promote well-being for children and their families. By identifying predictors of well-being, this paper contributed to the understanding of what was most important to a positive change in well-being during a pandemic. In preparation and planning for potential future pandemics, upstream efforts may be directed toward creating conditions conducive for family closeness (eg, communication and problem-solving skills). In conclusion, focusing on creating time adequacy for families to play, eat, and rest together may help improve well-being overall.
